# Evaluating disease burden in German AAV patients using the AAV-PRO: associations with disease activity, physical function, depression, fatigue and quality of life

**DOI:** 10.1186/s41687-026-01116-y

**Published:** 2026-06-10

**Authors:** Miriam Jacobs, Tim Filla, Gamal Chehab, Ayla Stütz, Amelie Niesmann, Marie Celine von Saan, Jutta G. Richter, Christina Düsing, Johanna Mucke, Nikolas Ruffer, Oliver Sander, Matthias Schneider, Michael Schmitz, Anna Kernder

**Affiliations:** 1 Department of Rheumatology, University Hospital Duesseldorf, Medical Faculty of Heinrich-Heine-University, Duesseldorf, Germany; 2 III. Department of Medicine, University Medical Center Hamburg-Eppendorf, Hamburg, Germany; 3 Städtisches Klinikum Solingen GmbH, Solingen, Germany; 4 Hiller Research Center, University Hospital Duesseldorf, Medical Faculty of Heinrich-Heine-University, Düsseldorf, Germany; 5 Rheumazentrum Ruhrgebiet Herne, Ruhr-University Bochum, Bochum, Germany; 6 Department of Rheumatology and Clinical Immunology, KEM Kliniken Essen-Mitte, Essen, Germany

**Keywords:** AAV-PRO, ANCA, Patient-reported outcome, Correlation, Vasculitis

## Abstract

**Background:**

The disease-specific patient-reported outcome for ANCA-associated vasculitis (AAV-PRO) was developed by the Outcome Measures in Rheumatology (OMERACT) group. Its interpretation in routine clinical practice remains challenging due to limited evidence regarding the association with established clinical outcome measures.

**Methodology:**

We conducted a prospective cohort study of 70 AAV-patients who completed the AAV-PRO and the following additional questionnaires: Short Form-36 (SF-36), assessing health-related quality of life; Fatigue Severity Scale (FSS) measuring fatigue severity; *Index zur Messung von Einschränkungen der Teilhabe* (IMET), assessing restrictions in participation in everyday life; *Funktionsfragebogen-Hannover* (FFbH), evaluating abilities and limitations in daily activities; Patient Health Questionnaire-9 (PHQ-9), screening for depressive symptoms; and Life Orientation Test–Revised (LOT-R), assessing optimism and expectations. Physicians assessed disease activity, related damage and glucocorticoid side-effects using the Birmingham Vasculitis Activity Score (BVAS), vasculitis damage index (VDI) and glucocorticoid toxicity index (GTI).

**Results:**

70 patients with AAV participated in the study, including 45 patients with GPA; 64.3%, 16 with EGPA; 22.9%, and 9 with MPA; 12.8%. At study inclusion, the mean disease duration was 10.5 years (SD 7.9). Patients with active disease had higher AAV-PRO scores across all domains. We observed strong correlations between AAV-PRO subdomains and the following validated measures: “Systemic symptoms” with SF-36 physical function (r = –0.72, 95% CI -0.85 to -0.58) and FFbH (r = –0.88, 95% CI -0.56 to -0.97); “Social and emotional impact” with SF-36 social role functioning (r = –0.8, 95% CI -0.87 to -0.69), IMET (r = 0.7, 95% CI 0.55 to 0.81) and FFbH (r = -0.75, 95% CI -0.94 to -0.23); “Concerns about the future” with SF-36 social role functioning (r = –0.71, 95% CI -0.81 to -0.55) and FFbH (r = –0.77, 95% CI -0.94 to -0.27); “Physical function” with SF-36 physical function (r = –0.9, 95% CI -0.94 to -0.84) and IMET (r = 0.79, 95% CI 0.68 to 0.87).

**Conclusion:**

The German AAV-PRO captures patient-perceived disease burden, complementing traditional clinical outcome measures. Its multidimensional approach allows assessment of functional, psychosocial and symptom-related aspects of AAV, supporting its use in clinical practice.

**Supplementary Information:**

The online version contains supplementary material available at 10.1186/s41687-026-01116-y.

## Introduction

ANCA-associated vasculitides (AAV) are rare, potentially life-threatening inflammatory diseases involving small- to medium-sized blood vessels characterized by a heterogeneous clinical presentation and a relapsing–remitting disease course [1, 2]. While disease activity and organ involvement are central targets of clinical management, they do not fully capture the individual burden experienced by the patients [3, 4]. Symptoms such as fatigue, pain, physical limitations, and emotional distress may persist despite controlled disease activity and are often insufficiently addressed by physician-reported measures [3]. In this context, patient-reported outcomes (PROs) provide a valuable tool to assess the severity and impact of AAV from the patient’s perspective, enabling a more comprehensive evaluation of disease burden and supporting patient-centered care and shared therapeutic decision-making [5].

The ANCA-associated Vasculitis Patient-Reported Outcome (AAV-PRO) was developed within the Outcome Measures in Rheumatology (OMERACT) initiative to assess disease-specific patient-relevant domains, including disease-related symptoms, treatment-related side effects, physical functioning, and social and emotional impact [6].

Although a German translation of the AAV-PRO is available, its interpretation in routine clinical practice remains challenging due to limited data on its associations with established and validated outcome measures. Previous studies have primarily focused on comparisons with generic health-related quality of life instruments, such as the Short Form-36 (SF-36), and measures of depression [7]. While these domains are important, they do not fully capture the multidimensional impact of AAV on patients’ daily lives and the domains of the AAV-PRO.

To comprehensively understand the disease burden as reflected by the AAV-PRO, further evaluation of its associations with additional patient-reported domains is warranted. In particular, restrictions in participation in everyday life, which may be closely linked to social and emotional concerns, represent a relevant but underexplored aspect. Moreover, functional abilities and health-related limitations in daily activities are critical determinants of patients’ overall well-being [2]. Fatigue, a frequent and often debilitating symptom in patients with AAV, also contributes substantially to disease burden and should be considered in the interpretation of patient-reported outcomes [8].

The German-language version of the AAV-PRO has previously been formally validated by Niesmann et al. [9], including the assessment of test–retest reliability.

The aim of this study was to investigate the associations between the German AAV-PRO and validated measures of disease activity, physical function, participation, fatigue, depression, and health-related quality of life in patients with AAV, in order to support the interpretability and clinical utility of the AAV-PRO in German clinical practice.

## Methods

### Study design and participants

This prospective cohort study was conducted at the rheumatology outpatient clinic of the University Hospital Duesseldorf. A total of 70 patients were recruited. Eligible participants were adults (≥ 18 years) with a confirmed diagnosis of AAV, including granulomatosis with polyangiitis (GPA), eosinophilic granulomatosis with polyangiitis (EGPA), or microscopic polyangiitis (MPA), fulfilling the ACR/EULAR 2022 Classification Criteria [1].

### Patient-reported outcome measures

After providing written informed consent, participants completed the AAV-PRO questionnaire.

The AAV-PRO consists of 29 items grouped into six domains:

1. **Organ-specific symptoms** (5 items), assessing ear, nose, throat, ocular, and chest manifestations.
2. **Systemic symptoms** (4 items), addressing joint and muscle problems, fatigue, and fever.
3. **Treatment side effects** (5 items), including skin and gastrointestinal symptoms, as well as concerns regarding weight and appearance.
4. **Social and emotional impact** (6 items), assessing depressive symptoms, anxiety, and concentration difficulties.
5. **Concerns about the future** (5 items), covering issues related to independence in daily life and potential long-term consequences of the disease and its treatment.
6. **Physical function** (4 items), focusing on challenges and coping strategies in everyday activities.

In addition to the AAV-PRO, patients completed the following validated questionnaires: the Short Form-36 Health Survey (SF-36) [10], assessing health-related quality of life; the Fatigue Severity Scale (FSS) [11], measuring fatigue severity; the *Index zur Messung von Einschränkungen der Teilhabe* (IMET) [12], assessing restrictions in participation in everyday life; the German analogue to the Health assessment Questionnaire (HAQ) *Funktionsfragebogen Hannover* (FFbH) [13], evaluating functional abilities and limitations in daily activities; the Patient Health Questionnaire-9 (PHQ-9) [14], screening for depressive symptoms; and the Life Orientation Test–Revised (LOT-R) [15], assessing optimism and expectations regarding the future and social environment.

Additional patient-reported information included current disease activity and relapses (yes/no), date of symptom onset, and date of diagnosis.

### Physician-reported measures

Treating physicians completed standardized clinical assessments, including the Birmingham Vasculitis Activity Score (BVAS), version 3 [16], the Vasculitis Damage Index (VDI) [17] and the Glucocorticoid Toxicity Index (GTI) [18]. Information on current medication, organ manifestations, and disease relapses was also documented by the attending physicians.

Domains, scoring and interpretation of the questionnaires are presented in the Supplementary Material Table S3.

### Study procedures

At baseline (T1), all participants and their treating physicians completed the respective questionnaires. Follow-up assessments (T2) were conducted after 3 to 6 months during routine outpatient visits, at which time patients and physicians were asked to complete the same set of questionnaires. The timing of follow-up assessments (3 or 6 months) depended on the individual clinical course, disease activity, and treatment modifications. Relapse was defined as the acute occurrence of new symptoms or worsening of pre-existing symptoms, as assessed by the treating physician.

### Ethical approval

The study was conducted in accordance with the Declaration of Helsinki and Good Clinical Practice guidelines. Ethical approval was obtained from the Ethics Committee of Heinrich-Heine-University Duesseldorf (file number 2019 − 602). All participants provided written informed consent prior to study participation.

### Statistical analysis

Statistical analyses were performed using R (version 4.3). Descriptive statistics for all patient-reported and physician-reported outcome measures were calculated and are presented as mean and standard deviation. To allow comparison across instruments with different scoring ranges, questionnaire scores were transformed to a standardized 0–100 scale, with higher values indicating better health status. Instruments in which lower scores represent better outcomes were inverted prior to transformation.

To investigate how the German AAV-PRO and its subdomains relate to established patient-reported and physician-reported outcome measures to address the question of which constructs are captured by the AAV-PRO in routine clinical assessment, we conducted a cross-sectional analysis at T1 and T2. As data between T1 and T2 did not show relevant differences, we present data from T1 within the manuscript and included the follow-up data from time point T2 in the Supplementary Material (Table S2).

Associations between binary variables and continuous variables were examined using the Kruskal–Wallis test followed by Dunn’s post hoc test where appropriate. Correlations between continuous variables are presented using Pearson’s correlation coefficients. As a sensitivity analysis, correlations were compared with Spearman correlation coefficients and presented in the Supplementary Material (Table S1). To facilitate interpretation of the AAV-PRO in clinical practice, correlations were analysed separately for each AAV-PRO subdomains with validated patient-reported and physician-reported outcome measures.

To facilitate interpretation of the correlation analyses, effect sizes were interpreted according to commonly used recommendations, with correlation coefficients of 0.00–0.19 considered very weak, 0.20–0.39 weak, 0.40–0.59 moderate, 0.60–0.79 strong, and ≥ 0.80 very strong correlations [19].

For each AAV-PRO domain, established questionnaires were selected that capture overlapping domains, including disease-activity, physical functioning, participation in everyday life, fatigue, emotional well-being, and treatment-related impairment.

## Results

### Study population

In total, 70 patients with AAV participated in the study, including 45 patients with GPA (64.3%), 16 with EGPA (22.9%) and 9 with MPA (12.8%). Of the participants, 34 (48.5%) self-identified as female and 36 (51.5%) as male. The mean age was 60.1 years (SD 13.7). At study inclusion, the mean disease duration was 126 months (SD 118.5). Based on patient self-report, 27 (38.5%) reported at least one relapse within the preceding two years. According to physician assessment, 14 (20%) experienced a relapse during the same period (Table 1).

**Table 1:** Description of the study cohort

		Study cohort (*n* = 70)
EGPA GPA MPA		16 (22.9%) 45 (64.3%) 9 (12.8%)
female male mean age (SD)		34 (48.57%) 36 (51.42%) 60.1 years (13.7)
Active disease (according to physician BVAS > = 1)	Yes No No statement	16 (22.8%) 22 (31.4%) 32 (45.7%)
Active disease according to patient	Yes No No statement	21 (30%) 44 (62.8%) 5 (7.1%)
Organ involvement	Kidney Lungs ENT Skin PNS CNS Cor	22 (31.4%) 29 (41.4%) 36 (51.4%) 8 (11.4%) 23 (32.8%) 7 (10%) 8 (11.4%)
Medication due to vasculitis	Rituximab Methotrexat Azathioprin Mycophenolat Mofetil Mepolizumab Cortison Cotrimoxazol	20 (28.5%) 11 (15.7%) 11 (15.7%) 4 (5.7%) 7 (10%) 24 (34.3%) 5 (7.1%)
	No medication	15 (21.4%)
	> 1 medication	22 (31.4%)

GPA: granulomatosis with polyangiitis, EGPA: eosinophilic granulomatosis with polyangiitis, MPA: microscopic polyangiitis; BVAS: Birmingham Vasculitis Activity Index; ENT: ear-nose-throat; PNS: peripheral nervous system; CNS: central nervous system

Agreement between patient- and physician-reported disease state (active vs. non-active) was 60%. Patients with self-reported active disease demonstrated consistently higher AAV-PRO scores across all domains compared with the overall study population. Patients reporting an active disease state showed markedly higher mean scores in systemic symptoms (32.81 vs. 9.82, *p* < 0.001), social/emotional impact (50.00 vs. 6.01, *p* < 0.001), concerns about the future (56.25 vs. 7.64, *p* < 0.001), and physical function impairment (32.8 vs. 5.09, *p* < 0.001). In addition, these patients demonstrated higher fatigue burden measured by the FSS (41.75 vs. 35.27).

At baseline (T1), 24 patients (34.3%) received glucocorticoid therapy, with a mean daily dose of 4.6 mg (SD 2.9). During the study period, 24 patients (34.3%) experienced changes in their medication regimen. Among patients receiving glucocorticoids, the dose was reduced in 14 cases (58.3%) and increased in 2 cases (8.3%). The mean interval between study visits was 168.3 days (SD 73.6).

**Fig. 1:**
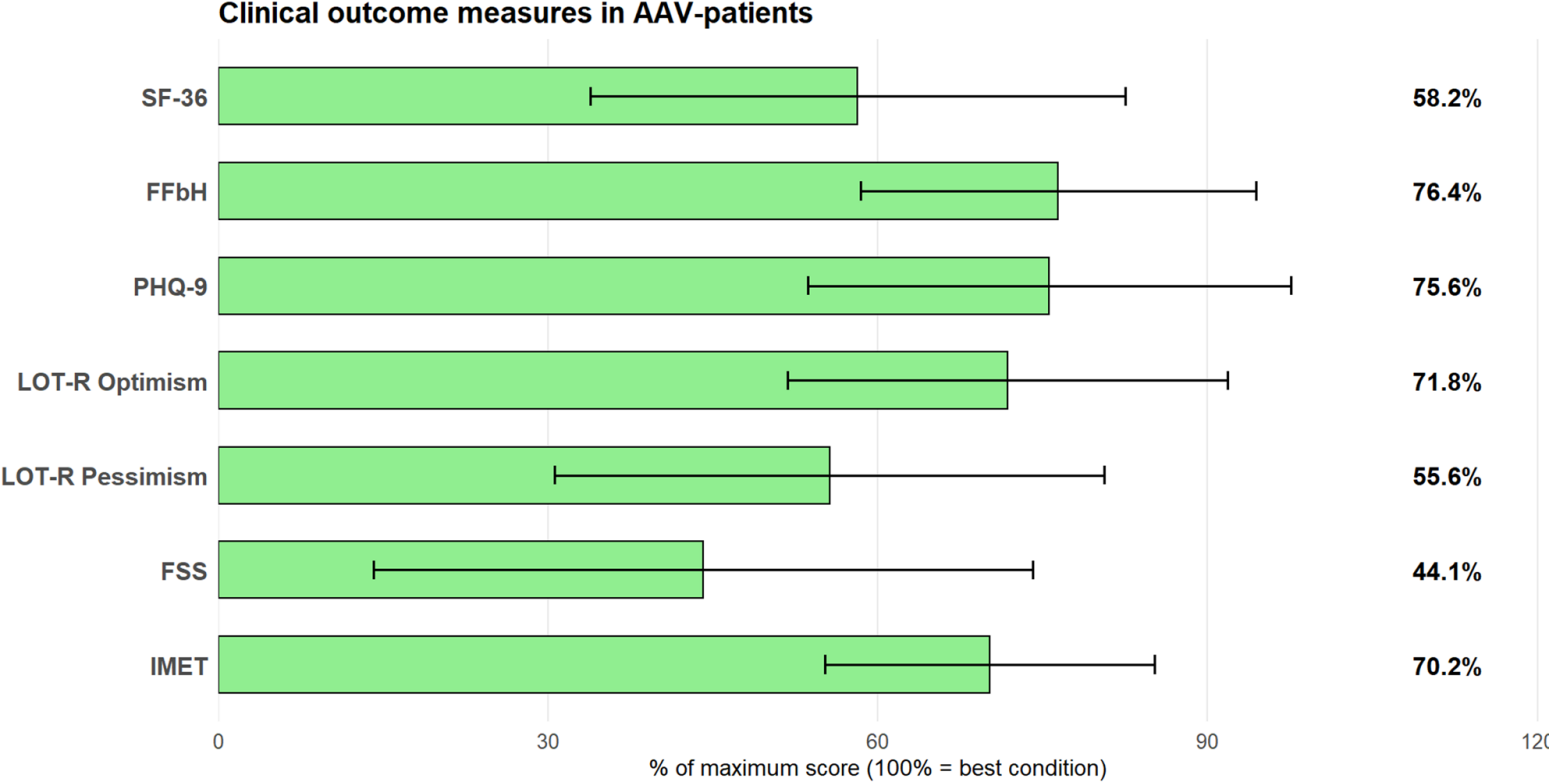
Clinical outcome measures in AAV-patients. SF-36 (Short Form-36), FFbH (Funktionsfragebogen Hannover, PHQ-9 (Patiens-health questionnaire 9), LOT-R (Life Orientation Test-Revised), FSS (Fatigue Severity Score), IMET (Index zur Messung von Einschränkungen der Teilhabe)

Figure 1 presents the disease burden according to measured established PROs. To allow comparison across instruments with different scoring ranges, questionnaire scores were transformed to a standardized 0–100 scale, with higher values indicating better health status.

### Associations between AAV-PRO domains and established outcome measures

**Fig. 2:**
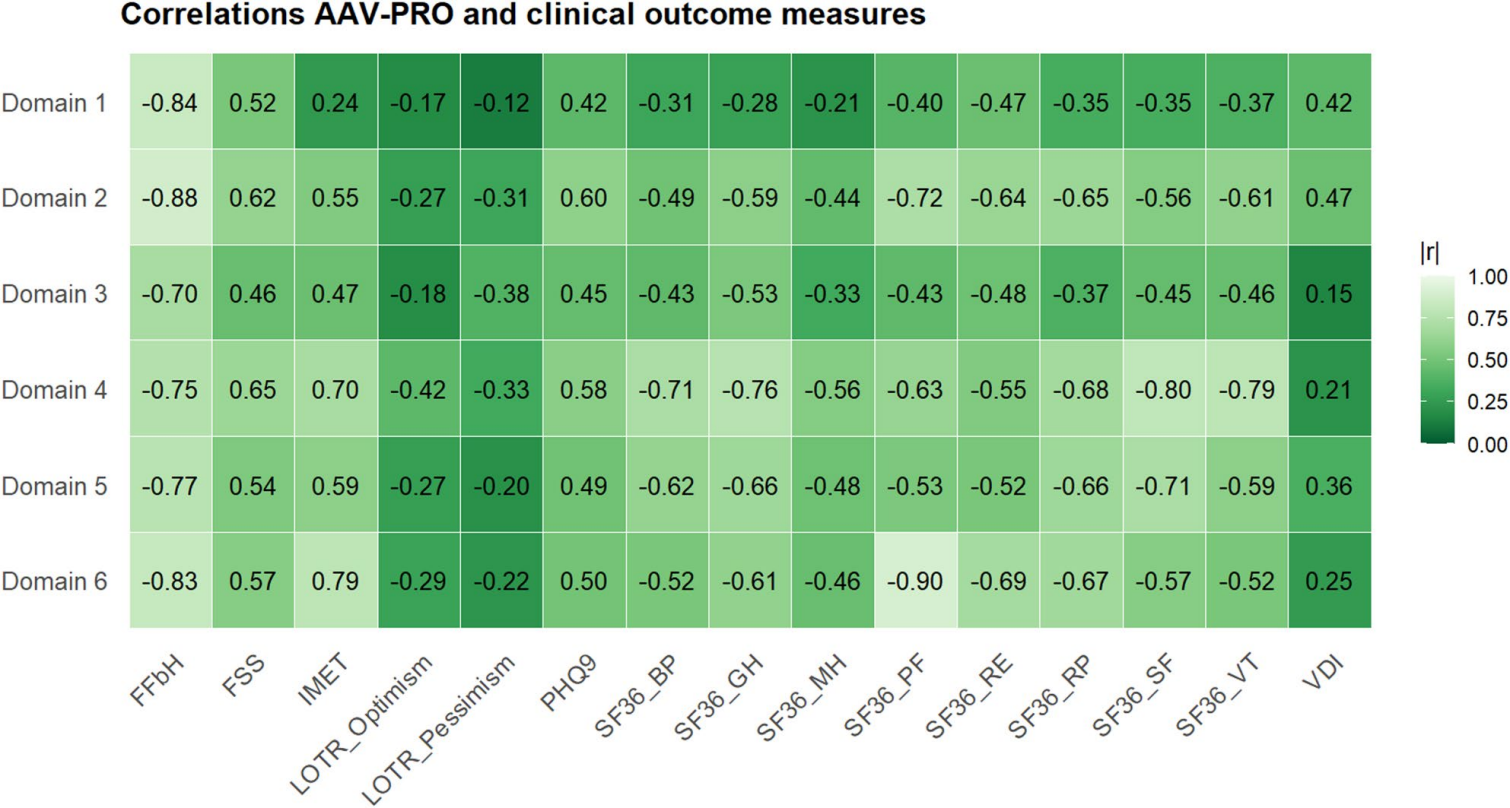
Heatmap: correlations of AAV-PRO and clinical outcome measures. The intensity of the green color represents the strength of the correlation coefficients, with lighter shades indicating stronger correlations and darker shades indicating weaker correlations, Domain 1: “organ specific symptoms”, Domain 2: “systemic symptoms”, Domain 3: “treatment side effects”, Domain 4: “social and emotional impact”, Domain 5: “concerns about the future”, Domain 6: “physical function”; SF-36: physical function (PF), role limitations due to physical health (RP), bodily pain (BP), general health perceptions (GH), vitality (VT), social role functioning (SF), role limitations due to emotional health (RE), mental health (MH); FSS (Fatigue Severity Score); PHQ-9 (Patient Health Questionnaire 9), LOT-R (Life Orientation Test-Revised), FFbH (Funktionsfragebogen Hannover) IMET (Index zur Messung von Einschränkungen der Teilhabe), VDI (Vasculitis Damage Index)

Figure 2 presents a heatmap illustrating the correlations between AAV-PRO domains and external clinical outcome measures. Overall, statistically significant strong associations were observed between all AAV-PRO domains and functional abilities and limitations in daily activities (FFbH: *r*≥-0.7), a moderate association (r 0.42–0.60) was found between the AAV-PRO domains and depressive symptoms, measured by the PHQ-9, Fig. 2 and Table 2.

**Table 2:** Results of the questionnaires

Variable	Mean (Standard Deviation)
AAV-PRO Domain 1 AAV-PRO Domain 2 AAV-PRO Domain 3 AAV-PRO Domain 4 AAV-PRO Domain 5 AAV-PRO Domain 6	11.71 (22.6) 9.82 (21.1) 7.64 (15.9) 6.01 (15.8) 7.64 (19.6) 5.09 (15.4)
FFbH	76.54 (20.84)
FSS	35.27 (19.04)
IMET	29.76 (23.71)
LOT-R Optimism LOT-R Pessimism	8.57 (2.44) 6.63 (2.84)
PHQ-9	6.61 (5.24)
SF_36_PF SF_36_GH SF_36_VT SF_36_RE SF_36_RP SF_36_BP SF_36_MH SF_36_SF	61.52 (28.82) 46.07 (19.83) 48.57 (22.27) 60.71 (45.44) 44.20 (43.17) 66.21 (30.43) 68.00 (19.82) 71.20 (25.56)

AAV-PRO Domain 1: “organ specific symptoms”, Domain 2: “systemic symptoms”, Domain 3: “treatment side effects”, Domain 4: “social and emotional impact”, Domain 5: “concerns about the future”, Domain 6: “physical function”; FFbH (Funktionsfragebogen Hannover, IMET (Index zur Messung von Einschränkungen der Teilhabe), FSS (Fatigue Severity Score), LOT-R (Life Orientation Test-Revised), SF-36: physical function (PF), general health perceptions (GH), vitality (VT), role limitations due to emotional health (RE), role limitations due to physical health (RP), bodily pain (BP), mental health (MH), social role functioning (SF)

### Domain 1 – Organ-specific symptoms

**Fig. 3:**
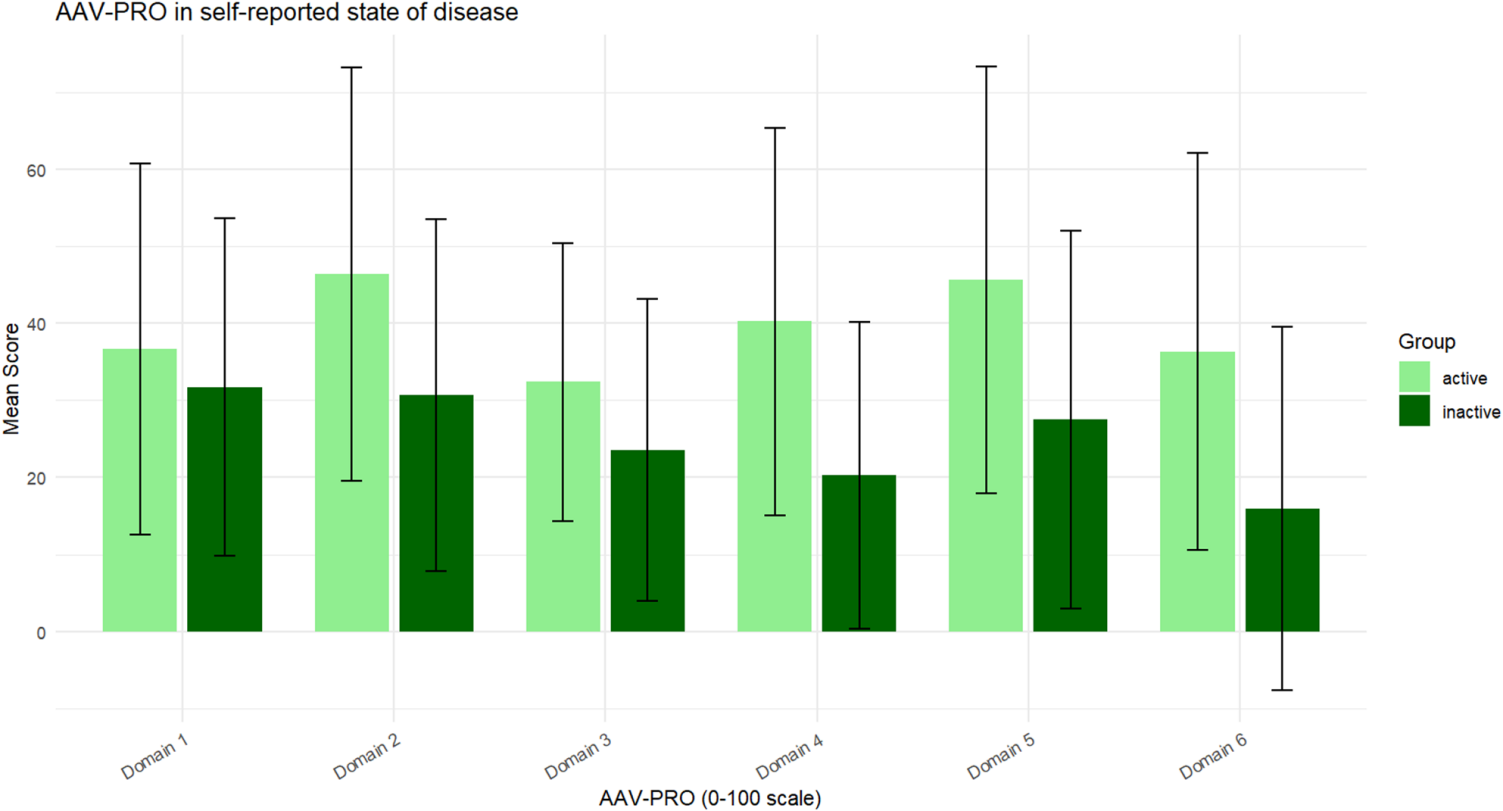
AAV-PRO scores depending on patient’s reported disease activity. AAV-PRO (0-100 scale): Domain 1: “organ specific symptoms”, Domain 2: “systemic symptoms”, Domain 3: “treatment side effects”, Domain 4: “social and emotional impact”, Domain 5: “concerns about the future”, Domain 6: “physical function”

**Fig. 4:**
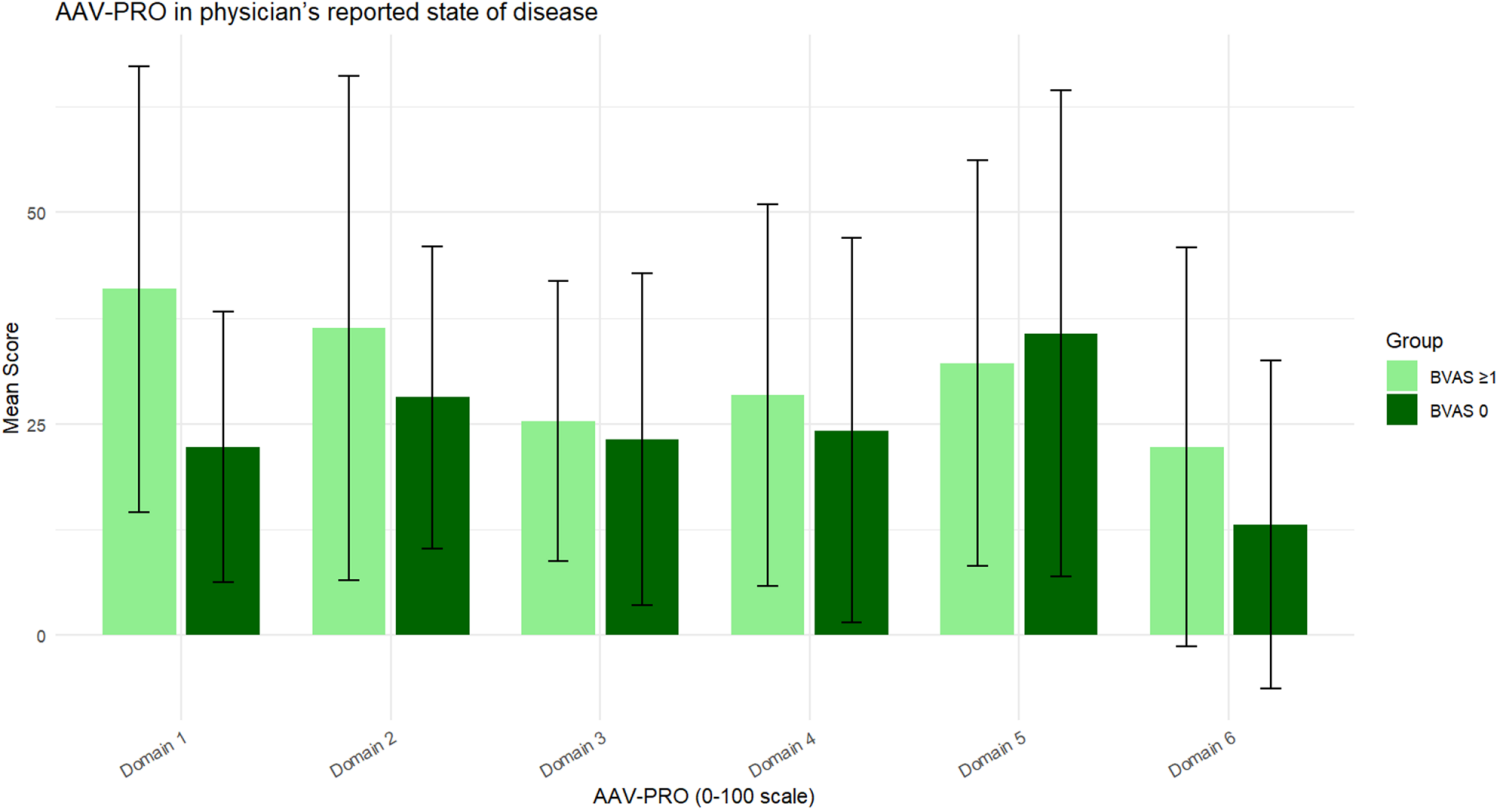
AAV-PRO scores depending on physician`s reported disease activity. BVAS = 0: inactive state of disease; BVAS > = 1: active state of disease; AAV-PRO (0-100 scale): Domain 1: “organ specific symptoms”, Domain 2: “systemic symptoms”, Domain 3: “treatment side effects”, Domain 4: “social and emotional impact”, Domain 5: “concerns about the future”, Domain 6: “physical function

The AAV-PRO domain “organ-specific symptoms” showed strong negative associations with the FFbH (*r* = − 0.84, 95% CI -0.96 to -0.45), indicating an overlap with limitations in everyday activities. Patients with active disease, defined by both self-report and BVAS ≥ 1 (physician reported), reported higher scores in this domain (mean self-reported: 36.43, CI 26.8 to 46.1, Fig. 3), compared with patients in remission (mean self-reported: 30.57, CI 24.6 to 36.5, Fig. 4).

### Domain 2 – Systemic symptoms

Strong associations were observed between the “systemic symptoms” domain and instruments capturing physical and psychological burden, including SF-36 physical functioning (*r* = − 0.72, 95% CI -0.85 to -0.58) and FFbH (*r* = − 0.88, 95% CI -0.56 to -0.97). Although correlations with the Fatigue Severity Scale and PHQ-9 did not reach statistical significance, a trend toward higher fatigue scores was observed (*r* > 0.60) (Fig. 1). Again, patients with active disease, defined by both self-report and BVAS ≥ 1, reported higher scores in this domain (mean self-reported: 46.13, 95% CI 33.9 to 58.4, Fig. 3) compared with patients in remission (mean self-reported: 30.28, 95% CI 22.8 to 37.8, Fig. 4).

### Domain 3 – Treatment side effects

The “treatment side effects” domain showed no statistically significant association with the physician-reported Glucocorticoid Toxicity Index (GTI; *r* = − 0.08, 95% CI − 0.16 to 0.45) or Vasculitis Damage Index (VDI; *r* = 0.18, 95% CI -0.19 to 0.50). Among patient-reported measures, an association was observed with the FFbH score (*r* = − 0.70, 95% CI -0.13 to -0.92) (Fig. 2; Table 2).

### Domain 4 – Social and emotional impact

This domain demonstrated strong associations with multiple instruments assessing social participation and emotional well-being, including SF-36 social role functioning (*r* = − 0.8, 95% CI -0.87 to -0.69), IMET (*r* = 0.70, 95% CI 0.55 to 0.81) and FFbH (*r* = -0.75, 95% CI -0.94 to -0.23) (Fig. 2; Table 2).

### Domain 5 – Concerns about the future

Associations were observed with SF-36 “social role functioning” (*r* = − 0.71, 95% CI -0.81 to -0.55) and FFbH (*r* = − 0.77, 95% CI -0.94 to -0.27), both addressing limitations in everyday and social contexts (Fig. 2; Table 2).

### Domain 6 – Physical function

The “physical function” domain correlated with the SF-36 physical function (*r* = − 0.90, 95% CI -0.94 to -0.84) and IMET (*r* = 0.79, 95% CI 0.68 to 0.87), reflecting overlap with limitations in daily activities (Fig. 2; Table 2).

No relevant associations were observed between the AAV-PRO scores and the Fatigue Severity Scale (all < 0.39) and the Life Orientation Test–Revised (all > 0.43) (Fig. 2).

## Discussion

In this study, we investigated the use of the German version of the AAV-PRO in a cohort of 70 patients with AAV and examined its association with established patient- and physician-reported outcome measures, thereby addressing the question of which constructs are captured by the AAV-PRO in routine clinical assessment.

While previous studies by Maunz et al. [7] and Niesmann et al. [9] primarily evaluated the psychometric validity and reliability of the German AAV-PRO, the present study focused on the clinical interpretation of the questionnaire domains. Specifically, we aimed to determine which dimensions of disease burden are reflected by the individual AAV-PRO domains through comparison with established instruments assessing physical function, participation restrictions, fatigue, depressive symptoms, and health-related quality of life, including the FFbH, IMET, LOT-R and FSS.

Our findings indicate that the AAV-PRO captures multiple aspects of disease burden that are relevant to patients’ everyday lives.

### Relevance of patient-reported outcomes in AAV

AAV is a heterogeneous disease with fluctuating activity and diverse organ involvement, making comprehensive assessment challenging [5]. Traditional physician-based outcome measures, such as BVAS and VDI, provide valuable clinical information but may not fully reflect the patients’ perspective [3–5].

Our results highlight the added value of patient-reported outcomes: while agreement between patient- and physician-reported disease activity was only 60%, patients reported higher scores in all AAV-PRO domains during self-reported active disease. Patients with self-reported active disease demonstrated consistently higher AAV-PRO scores across all domains compared with the overall study population. Patients reporting active disease showed markedly higher mean scores in systemic symptoms (32.81 vs. 9.82, *p* < 0.001), social/emotional impact (50.00 vs. 6.01, *p* < 0.001), concerns about the future (56.25 vs. 7.64, *p* < 0.001), and physical function impairment (32.8 vs. 5.09, *p* < 0.001). In addition, these patients demonstrated higher fatigue burden measured by the FSS (41.75 vs. 35.27).

In contrast, the total BVAS score did not strongly correlate with several AAV-PRO domains, in line with previous reports [6, 7, 9]. These findings suggest that patients who perceive their disease as active experience substantial residual disease burden even when physician-assessed activity is low.

Rohde et al. demonstrated that patient and physician assessments are influenced by different determinants [20]. Physician-assessed disease activity was primarily associated with objective inflammatory markers such as CRP levels and disease duration, whereas patient-reported disease activity was more strongly associated with pain, functional limitations in daily living, and impaired physical well-being. Furthermore, Rohde et al. demonstrated only a moderate correlation between physician and patient global assessments (Pearson *r* = 0.31), while patients were more likely to report higher disease activity scores than physicians, similar to our study cohort.

The observed discordance between physician- and patient-reported disease activity suggests that conventional physician-based activity measures may underestimate relevant residual disease burden experienced by patients. While physician assessments primarily reflect inflammatory activity and organ manifestations, patient-reported burden is additionally influenced by pain, fatigue, psychosocial limitations, physical functioning, and treatment-related effects. Consequently, the AAV-PRO may provide complementary information regarding ongoing patient-relevant impairment, even in patients considered to be in clinical remission according to BVASv3.

These findings are supported by a recent cross-sectional study demonstrating moderate correlations between several AAV-PRO domains and patient global assessment, while physician global assessment showed different associations, mainly reflecting clinical and treatment-related parameters. The authors further identified differences in AAV-PRO domain scores according to sex, age, and disease duration, underlining the multidimensional and patient-specific nature of disease burden in AAV [21].

### Domain-specific insights

By examining the AAV-PRO domains separately, we were able to relate each domain to validated outcome measures, strengthening the interpretability of the instrument in clinical practice and its concomitant use in addition to other PROs.

Correlation patterns between AAV-PRO domains and external validation instruments were broadly comparable across T1 and T2, with consistent directions and similar effect sizes over time. Particularly stable associations were observed for SF-36, FSS, IMET, and FFbH measures, supporting the longitudinal robustness of the construct validity of the AAV-PRO.

Strong associations between the domains “organ-specific symptoms” and “systemic symptoms” and measures of physical functioning, including SF-36 physical functioning and FFbH, demonstrate the close relationship between disease manifestations and limitations in daily functioning. Together, these findings emphasize that organ-specific and systemic symptoms in AAV substantially affect patients’ daily functioning and perceived health status.

The domain “social and emotional impact” demonstrated strong correlations with SF-36 social functioning, IMET, and FFbH, underlining the substantial psychosocial burden experienced by patients with AAV. Similarly, the domain “concerns about the future” was associated with measures of social participation and functional capacity, indicating that future-related concerns are closely linked to patients’ current physical and social limitations.

The very strong correlation between the AAV-PRO physical function domain and the SF-36 physical functioning subscale further supports the convergent validity and clinical interpretability of the questionnaire. In addition, strong associations with FFbH and IMET demonstrate that this domain captures relevant limitations in daily activities and participation.

For the domain “treatment side effects”, only weak associations with physician-reported toxicity measures such as GTI and VDI were observed. This finding may indicate that patient-perceived treatment burden differs from physician-derived toxicity assessments. While the GTI focuses on objectively assessable glucocorticoid-related toxicities, the AAV-PRO may reflect a broader and more subjective perception of treatment impact.

Furthermore, the GTI is influenced by disease duration and cumulative glucocorticoid exposure, whereas the AAV-PRO does not explicitly account for cumulative medication burden. As cumulative glucocorticoid dose was not assessed in our study, conclusions regarding long-term treatment-related effects remain limited.

Therefore, the ability of the AAV-PRO to comprehensively assess treatment-related side effects should be interpreted with caution.

### Clinical implications

These results support the use of the AAV-PRO alongside traditional physician-reported assessments and established patient-reported measures. While certain domains, particularly physical functioning, show substantial overlap with established generic instruments, the AAV-PRO additionally captures disease-specific psychosocial burden, treatment-related concerns, and future-related worries that are not comprehensively represented in generic quality-of-life measures.

At the same time, our findings highlight potential limitations of the AAV-PRO in assessing cumulative toxicity and irreversible organ damage.

The lack of statistically significant associations with the PHQ-9 and FSS may indicate potential limitations of the AAV-PRO regarding the assessment of depressive symptoms and fatigue. In particular, fatigue has consistently been identified as a major determinant of impaired quality of life and daily functioning in AAV patients [8]. However, exploratory item-level analyses demonstrated a moderate positive association between FSS and AAV-PRO question 9 (*r* = 0.403, *p* < 0.001), suggesting that aspects of fatigue-related burden are represented within the questionnaire. Since correlations between the AAV-PRO and dedicated fatigue measures have not previously been investigated in detail, this aspect warrants further evaluation in future studies.

In addition, recent prospective data in patients with EGPA treated with mepolizumab demonstrated that the AAV-PRO is sensitive to early changes in health-related quality of life, with statistically significant improvements observed within days to weeks after treatment initiation. Particularly strong improvements were reported in organ-specific symptoms and physical function domains, supporting the responsiveness and potential clinical utility of the AAV-PRO for monitoring patient-reported treatment benefit in routine care [22].

### Comparison with previous studies

Our findings are consistent with earlier validation studies comparing the AAV-PRO with disease activity and SF-36 [7, 23, 24], but extend them by including additional measures such as FFbH, IMET, PHQ-9, FSS, VDI and GTI to capture limitations in daily functioning, participation, cumulative organ damage, and treatment-related toxicity.

Furthermore, the absence of statistically significant correlations with the VDI and GTI may indicate limited ability of the AAV-PRO to reflect cumulative organ damage and glucocorticoid-related toxicity. This underlines the value of the questionnaire as a complementary patient-reported measure that captures subjective disease burden in addition to objective clinical assessment tools.

When comparing the study cohorts, differences in mean disease duration become apparent. In both our study and the original publication by Robson et al. [6], patients had a relatively long mean disease duration (126 months and 111.6 months, respectively), whereas shorter disease durations were reported by Maunz et al. and Monti et al. (56.5 and 70 months, respectively) [7, 24]. Despite these differences, all studies reached broadly comparable conclusions regarding the validity and applicability of the AAV-PRO.

### Limitations

The study was conducted at a single tertiary care center, which may limit the generalizability of the findings. Due to the rarity of AAV, the cohort size was relatively small (*n* = 70), particularly within the MPA subgroup. However, comparable cohort sizes have been reported in other single-center studies investigating patient-reported outcomes in AAV, such as Monti et al. [24]. Nevertheless, the limited sample size may have reduced statistical power and requires cautious interpretation of weaker or statistically non-significant associations. Patients with uncertain or unclassified diagnoses were excluded in order to maintain a well-defined AAV cohort, although this may additionally limit generalizability.

In addition, given the exploratory nature of this study and the large number of correlation analyses performed, the risk of type I error due to multiple testing cannot be excluded and should be considered when interpreting the findings.

Furthermore, the mean disease duration in our cohort was 126 months, indicating a population with longstanding disease. Consequently, accumulated disease burden and irreversible organ damage may have influenced domains such as physical function and concerns about the future.

### Conclusion

The German version of the AAV-PRO provides a clinically interpretable measure of patient-reported burden in AAV and captures psychosocial, functional, and symptom-related aspects relevant to patients with AAV. Its use in routine clinical practice alongside other patient-reported and physician-reported outcome measures may support patient-centered care and shared therapeutic decision-making.

## Supplementary Information

Below is the link to the electronic supplementary material.

Supplementary Material 1

## Data Availability

No datasets were generated or analysed during the current study.
