# Supplementary material for: Evaluating disease burden in German AAV patients using the AAV-PRO: associations with disease activity, physical function, depression, fatigue and quality of life
Source: J Patient Rep Outcomes. 2026 Jun 10;10:99. doi: 10.1186/s41687-026-01116-y (PMC13260533; doi:10.1186/s41687-026-01116-y)
Supplement: Supplementary file 1 — Supplementary Material 1 [file 41687_2026_1116_MOESM1_ESM.docx]

**Supplementary material**

**Supplementary Table 1. Comparison of Pearson and Spearman correlation coefficients between AAV-PRO domains and external validation questionnaires as sensitivity analysis.**

|  |  |  | | |  | | **AAV-PRO** | |  | |  | |  |
| --- | --- | --- | --- | --- | --- | --- | --- | --- | --- | --- | --- | --- | --- |
| Questionnaire | Test | | Organ Specific Symptoms | Systemic Symptoms | | Treatment side effects | | Social and Emotional Impact | | Concerns about the Future | | Physical Function |  |
| SF36_mcs | Spearman | | -0.373 | -0.710 | | -0.400 | | -0.630 | | -0.519 | | -0.916 |  |
|  | Pearson | | -0.45 | -0.72 | | -0.54 | | -0.83 | | -0.72 | | -0.72 |  |
| SF36_pcs | Spearman | | -0.333 | -0.657 | | -0.394 | | -0.707 | | -0.662 | | -0.748 |  |
|  | Pearson | | -0.39 | -0.7 | | -0.51 | | -0.81 | | -0.72 | | -0.76 |  |
| SF36_PF | Spearman | | -0.373 | -0.71 | | -0.4 | | -0.63 | | -0.519 | | -0.91 |  |
|  | Pearson | | -0.4 | -0.72 | | -0.43 | | -0.63 | | -0.53 | | -0.9 |  |
| SF36_RP | Spearman | | -0.333 | -0.657 | | -0.394 | | -0.707 | | -0,662 | | -0.74 |  |
|  | Pearson | | -0.35 | -0.65 | | -0.37 | | -0.68 | | -0.66 | | -0.67 |  |
| SF36_BP | Spearman | | -0.44 | -0.68 | | -0.445 | | -0.503 | | -0.493 | | -0.64 |  |
|  | Pearson | | -0.31 | -0.49 | | -0.43 | | -0.71 | | -0.62 | | -0.52 |  |
| SF36_GH | Spearman | | -0.211 | -0.47 | | -0.398 | | -0.608 | | -0.52 | | -0.52 |  |
|  | Pearson | | -0.28 | -0.59 | | -0.53 | | -0.76 | | -0.66 | | -0.61 |  |
| SF36_VT | Spearman | | -0.239 | -0.588 | | -0.523 | | -0.762 | | -0.62 | | -0.628 |  |
|  | Pearson | | -0.37 | -0.61 | | -0.46 | | -0.79 | | -0.59 | | -0.52 |  |
| SF36_SF | Spearman | | -0.323 | -0.544 | | -0.484 | | -0.809 | | -0.725 | | -0.63 |  |
|  | Pearson | | -0.35 | -0.56 | | -0.45 | | -0.8 | | -0.71 | | -0.57 |  |
| SF36_RE | Spearman | | -0.26 | -0.439 | | -0.389 | | -0.687 | | -0.58 | | -0.518 |  |
|  | Pearson | | -0.47 | -0.64 | | -0.48 | | -0.55 | | -0.52 | | -0.69 |  |
| SF36_MH | Spearman | | -0.236 | -0.502 | | -0.479 | | -0.74 | | -0.55 | | -0.45 |  |
|  | Pearson | | -0.21 | -0.44 | | -0.33 | | -0.56 | | -0.48 | | -0.46 |  |
| LOTR_opt | Spearman | | -0.198 | -0.275 | | -0.275 | | -0.422 | | -0.32 | | -0.31 |  |
|  | Pearson | | -0.17 | -0.27 | | -0.18 | | -0.42 | | -0.27 | | -0.29 |  |
| LOTR_pes | Spearman | | -0.088 | -0.328 | | -0.03 | | -0.236 | | -0.208 | | -0.2 |  |
|  | Pearson | | -0.12 | -0.31 | | -0.38 | | -0.33 | | -0.2 | | -0.22 |  |
| IMET | Spearman | | 0.356 | 0.73 | | 0.312 | | 0.599 | | 0.51 | | 0.721 |  |
|  | Pearson | | 0.24 | 0.55 | | 0.47 | | 0.7 | | 0.59 | | 0.79 |  |
| FFBH | Spearman | | -0.85 | -0.876 | | -0.59 | | -0.699 | | -0.786 | | -0.82 |  |
|  | Pearson | | -0.84 | -0.88 | | -0.7 | | -0.75 | | -0.77 | | -0.83 |  |
| VDI | Spearman | | 0.39 | 0.477 | | 0.224 | | 0.280 | | 0.493 | | 0.405 |  |
|  | Pearson | | 0.42 | 0.47 | | 0.15 | | 0.21 | | 0.36 | | 0.25 |  |
| FSS | Spearman | | 0,215 | 0.471 | | 0.208 | | 0.437 | | 0.353 | | 0.311 |  |
|  | Pearson | | 0.24 | 0.39 | | 0.21 | | 0.4 | | 0.35 | | 0.38 |  |
| BVAS_V3 | Spearman | | 0.402 | 0.08 | | 0.09 | | 0,119 | | -0.03 | | 0.223 |  |
|  | Pearson | | 0.49 | 0.33 | | 0.2 | | 0.27 | | 0.05 | | 0.3 |  |
| PHQ-9 | Spearman | | 0.334 | 0.578 | | 0.376 | | 0.551 | | 0.476 | | 0.491 |  |
|  | Pearson | | 0.42 | 0.60 | | 0.45 | | 0.58 | | 0.49 | | 0.508 | |

SF-36 (Short Form-36 Health Survey): physical function (PF), role limitations due to physical health (RP), bodily pain (BP), general health perceptions (GH), vitality (VT), social role functioning (SF), role limitations due to emotional health (RE), mental health (MH); FSS (Fatigue Severity Score); PHQ-9 (Patient Health Questionnaire 9), LOT-R (Life Orientation Test-Revised, with opt optimism and pes pessimism subscale), FFbH (Funktionsfragebogen Hannover) IMET (Index zur Messung von Einschränkungen der Teilhabe), VDI (Vasculitis Damage Index), BVAS_V3 (Birmingham Vasculitis activity Score, version 3)

**Supplementary Table 2. Comparison of correlations between AAV-PRO domains and external validation questionnaires at baseline (t1) and follow-up (t2)**

Correlation patterns between AAV-PRO domains and external validation instruments were broadly comparable across T1 and T2, with consistent directions and similar effect sizes over time. Particularly stable associations were observed for SF-36, FSS, IMET, and FFBH measures, supporting the longitudinal robustness of the construct validity of the AAV-PRO. Some variability was observed for VDI and LOT-R scores, potentially reflecting changes in disease burden or weaker conceptual overlap with patient-reported outcomes.

|  |  |  | | |  | | **AAV-PRO** | |  | |  | |
| --- | --- | --- | --- | --- | --- | --- | --- | --- | --- | --- | --- | --- |
| Questionnaire | Timepoint | | Organ Specific Symptoms | Systemic Symptoms | | Treatment side effects | | Social and Emotional Impact | | Concerns about the Future | | Physical Function |
| SF36_PF | t1 | | -0.4 | -0.72 | | -0.43 | | -0.63 | | -0.53 | | -0.9 |
|  | t2 | | -0.37 | -0.47 | | -0.34 | | -0.51 | | -0.53 | | -0.77 |
| SF36_RP | t1 | | -0.35 | -0.65 | | -0.37 | | -0.68 | | -0.66 | | -0.67 |
|  | t2 | | -0.5 | -0.54 | | -0.42 | | -0.59 | | -0.57 | | -0.69 |
| SF36_BP | t1 | | -0.31 | -0.49 | | -0.43 | | -0.71 | | -0.62 | | -0.52 |
|  | t2 | | -0.46 | -0.53 | | -0.4 | | -0.74 | | -0.73 | | -0.68 |
| SF36_GH | t1 | | -0.28 | -0.59 | | -0.53 | | -0.76 | | -0.66 | | -0.61 |
|  | t2 | | -0.45 | -0.49 | | -0.34 | | -0.61 | | -0.47 | | -0.53 |
| SF36_VT | t1 | | -0.37 | -0.61 | | -0.46 | | -0.79 | | -0.59 | | -0.52 |
|  | t2 | | -0.6 | -0.58 | | -0.47 | | -0.79 | | -0.71 | | -0.7 |
| SF36_SF | t1 | | -0.35 | -0.56 | | -0.45 | | -0.8 | | -0.71 | | -0.57 |
|  | t2 | | -0.37 | -0.44 | | -0.42 | | -0.75 | | -0.73 | | -0.63 |
| SF36_RE | t1 | | -0.47 | -0.64 | | -0.48 | | -0.55 | | -0.52 | | -0.69 |
|  | t2 | | -0.48 | -0.58 | | -0.38 | | -0.47 | | -0.41 | | -0.58 |
| SF36_MH | t1 | | -0.21 | -0.44 | | -0.33 | | -0.56 | | -0.48 | | -0.46 |
|  | t2 | | -0.27 | -0.42 | | -0.29 | | -0.55 | | -0.61 | | -0.57 |
| LOTR_opt | t1 | | -0.17 | -0.27 | | -0.18 | | -0.42 | | -0.27 | | -0.29 |
|  | t2 | | 0.01 | 0.03 | | 0.03 | | -0.25 | | -0.19 | | -0.22 |
| LOTR_pes | t1 | | -0.12 | -0.31 | | -0.38 | | -0.33 | | -0.2 | | -0.22 |
|  | t2 | | -0.01 | -0.1 | | -0.12 | | -0.06 | | 0.02 | | -0.19 |
| IMET | t1 | | 0.24 | 0.55 | | 0.47 | | 0.7 | | 0.59 | | 0.79 |
|  | t2 | | 0.47 | 0.6 | | 0.4 | | 0.72 | | 0.69 | | 0.82 |
| FFBH | t1 | | -0.84 | -0.88 | | -0.7 | | -0.75 | | -0.77 | | -0.83 |
|  | t2 | | -0.79 | -0.71 | | -0.67 | | -0.65 | | -0.71 | | -0.83 |
| VDI | t1 | | 0.42 | 0.47 | | 0.15 | | 0.21 | | 0.36 | | 0.25 |
|  | t2 | | 0.45 | 0.69 | | 0.63 | | 0.74 | | 0.67 | | 0.58 |
| FSS | t1 | | 0.24 | 0.39 | | 0.21 | | 0.4 | | 0.35 | | 0.38 |
|  | t2 | | 0.52 | 0.62 | | 0.46 | | 0.61 | | 0.54 | | 0.57 |
| BVAS_V3 | t1 | | 0.49 | 0.33 | | 0.2 | | 0.27 | | 0.05 | | 0.3 |
|  | t2 | | 0.23 | 0.45 | | 0.37 | | 0.43 | | 0.24 | | 0.34 |
| PHQ-9 | t1 | | 0.42 | 0.60 | | 0.45 | | 0.58 | | 0.49 | | 0.508 |
|  | t2 | | 0.551 | 0.5919 | | 0.500 | | 0.688 | | 0.584 | | 0.542 |

SF-36 (Short Form-36 Health Survey): physical function (PF), role limitations due to physical health (RP), bodily pain (BP), general health perceptions (GH), vitality (VT), social role functioning (SF), role limitations due to emotional health (RE), mental health (MH); FSS (Fatigue Severity Score); PHQ-9 (Patient Health Questionnaire 9), LOT-R (Life Orientation Test-Revised, with opt optimism and pes pessimism subscale), FFbH (Funktionsfragebogen Hannover) IMET (Index zur Messung von Einschränkungen der Teilhabe), VDI (Vasculitis Damage Index), BVAS_V3 (Birmingham Vasculitis activity Score, version 3)

**Supplementary Table 3. Overview of structure and interpretation of patient-reported and clinical outcome measures used**

| **Questionnaire** | | **Domains/outcome** | **Structure** | **Interpretation**  Higher scores indicate… |
| --- | --- | --- | --- | --- |
| AAV-PRO | 1. organ-specific symptoms 2. systemic symptoms 3. treatment side effects 4. social/emotional impact 5. concerns about the future 6. physical function | | 29 items across 6 domains | greater disease burden and impairment |
| SF-36 | 1. Physical functioning 2. role physical 3. bodily pain 4. general health 5. vitality 6. social functioning 7. role emotional 8. mental heath | | 8 subscales scored from 0–100 | better health-related quality of life |
| FSS | | Fatigue severity and impact on daily functioning | 9 items scored from 1–7; mean total score ranges from 1–7 | more severe fatigue |
| IMET | | Restrictions in participation and social functioning in daily life | 9 items scored from 0–10; total score ranges from 0–90 | greater impairment in participation |
| FFbH | | Functional ability in activities of daily living | Percentage score ranging from 0–100% | better functional status |
| PHQ-9 | | Depressive symptoms | 9 items scored from 0–3; total score ranges from 0–27 | greater depressive symptom burden |
| LOT-R | | Optimism and future expectations | 10 items (6 scored items + 4 filler items); total score ranges from 0–24 | Higher scores indicate greater dispositional optimism |
| BVAS v3 | | Physician-assessed vasculitis disease activity | Weighted organ-based activity score ranging from 0–63 | higher disease activity |
| VDI | | Accumulated irreversible organ damage | Damage items accumulated over time; total score ranges from 0 upward (no fixed maximum) | greater permanent damage |
| GTI | | Glucocorticoid-related toxicity | Composite toxicity score; aggregate improvement and worsening scores without a strict upper limit | greater glucocorticoid toxicity |

SF-36 (Short Form-36 Health Survey), FSS (Fatigue Severity Score); PHQ-9 (Patient Health Questionnaire 9), LOT-R (Life Orientation Test-Revised, with opt optimism and pes pessimism subscale), FFbH (Funktionsfragebogen Hannover) IMET (Index zur Messung von Einschränkungen der Teilhabe), VDI (Vasculitis Damage Index), BVAS_V3 (Birmingham Vasculitis activity Score, version 3), relevant references:

1. Robson, J.C., et al., *Validation of the ANCA-associated vasculitis patient-reported outcomes (AAV-PRO) questionnaire.* Ann Rheum Dis, 2018. 77(8): p. 1157-1164
2. Stewart, A.L., R.D. Hays, and J.E. Ware, Jr., *The MOS short-form general health survey. Reliability and validity in a patient population.* Med Care, 1988. 26(7): p. 724-35,
3. Valko, P.O. et al., *Validation of the fatigue severity scale in a Swiss cohort.* Sleep, 2008. 31(11): p. 1601-7.
4. *Deck R, Mittag O, Muche-Borowski C et al. Index zur Messung von Einschränkungen der Teilhabe (IMET) – Erste Ergebnisse eines ICF-orientierten Assessmentinstruments. Praxis Klinische Verhaltensmedizin und Rehabilitation 2007; 76: 113-120* Scheier, M.F., C.S. Carver, and M.W. Bridges, *Distinguishing optimism from neuroticism (and trait anxiety, self-mastery, and self-esteem): a reevaluation of the Life Orientation Test.* J Pers Soc Psychol, 1994. 67(6): p. 1063-78.
5. Haase I, Schwarz A, Burger A, Kladny B. Der Funktionsfragebogen Hannover (FFbH) und die Subskala "körperliche Funktionsfähigkeit" aus dem SF-36 im Vergleich [Comparison of Hannover Functional Ability Questionnaire (FFbH) and the SF-36 subscale "Physical Functioning"]. Rehabilitation (Stuttg). 2001 Feb;40(1):40-2. German. doi: 10.1055/s-2001-12127. PMID: 11253754.
6. Manea, L., S. Gilbody, and D. McMillan, *A diagnostic meta-analysis of the Patient Health Questionnaire-9 (PHQ-9) algorithm scoring method as a screen for depression.* Gen Hosp Psychiatry, 2015. 37(1): p. 67-75
7. Andersson, Gerhard. "The benefits of optimism: A meta-analytic review of the Life Orientation Test." *Personality and Individual Differences* 21.5 (1996): 719-725.Suppiah, R., et al., *A cross-sectional study of the Birmingham Vasculitis Activity Score version 3 in systemic vasculitis.* Rheumatology (Oxford), 2011. 50(5): p. 899-905.
8. Suppiah, R., et al., *A cross-sectional study of the Birmingham Vasculitis Activity Score version 3 in systemic vasculitis.* Rheumatology (Oxford), 2011. 50(5): p. 899-905.
9. Exley AR, Bacon PA, Luqmani RA et al. Development and initial validation of the vasculitis damage index for the standardized clinical assessment of damage in the systemic vasculitides. Arthritis Rheum 1997;40:371–80.
10. Miloslavsky EM, Naden RP, Bijlsma JW, Brogan PA, Brown ES, Brunetta P, Buttgereit F, Choi HK, DiCaire JF, Gelfand JM, Heaney LG, Lightstone L, Lu N, Murrell DF, Petri M, Rosenbaum JT, Saag KS, Urowitz MB, Winthrop KL, Stone JH. Development of a Glucocorticoid Toxicity Index (GTI) using multicriteria decision analysis. Ann Rheum Dis. 2017 Mar;76(3):543-546. doi: 10.1136/annrheumdis-2016-210002. Epub 2016 Jul 29. PMID: 27474764.
